# Supplementary material for: Patients with genetically heterogeneous synchronous colorectal cancer carry rare damaging germline mutations in immune-related genes
Source: Nat Commun. 2016 Jul 5;7:12072. doi: 10.1038/ncomms12072 (PMC4935966; doi:10.1038/ncomms12072)
Supplement: Supplementary Information — Supplementary Figures 1-14, Supplementary Tables 1-5, Supplementary Note 1 and Supplementary References. [file ncomms12072-s1.pdf]

Supplementary Figure 1. Estimation of tumour content

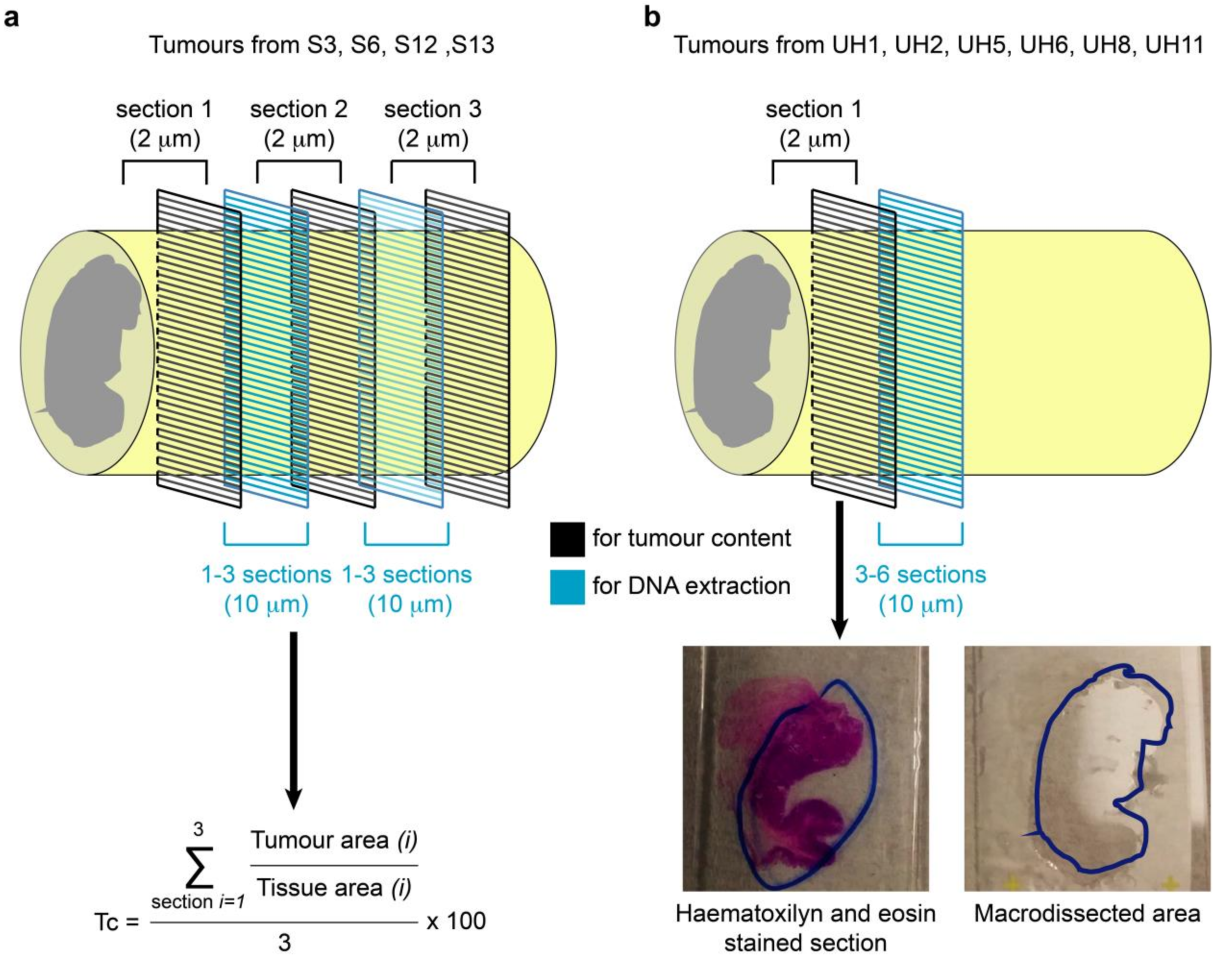

a, Approach used to estimate the tumour content in S13T1/T2, S6T1/T2, S3T1/T2 and S12T1/T2. Tissue and tumour areas were evaluated by two independent pathologist on three 2 μm-thick sections at the beginning, in the middle and at the end of the tissue block. The tumour content of these sections was calculated as the percentage of tumour area over the total tissue area.

b, Approach used to estimate the tumour content in UH1T1/T2, UH2T1/T2, UH5T1/T2, UH6T1/T2, UH8T1/T2 and UH11T1/T2. Two μm-thick FFPE section at the beginning of the block was stained for haematoxylin and eosin and the tumour area was delimited by pathologist. This was used as a reference to macrodissect the tumour.

Supplementary Figure 2. Sequencing throughput

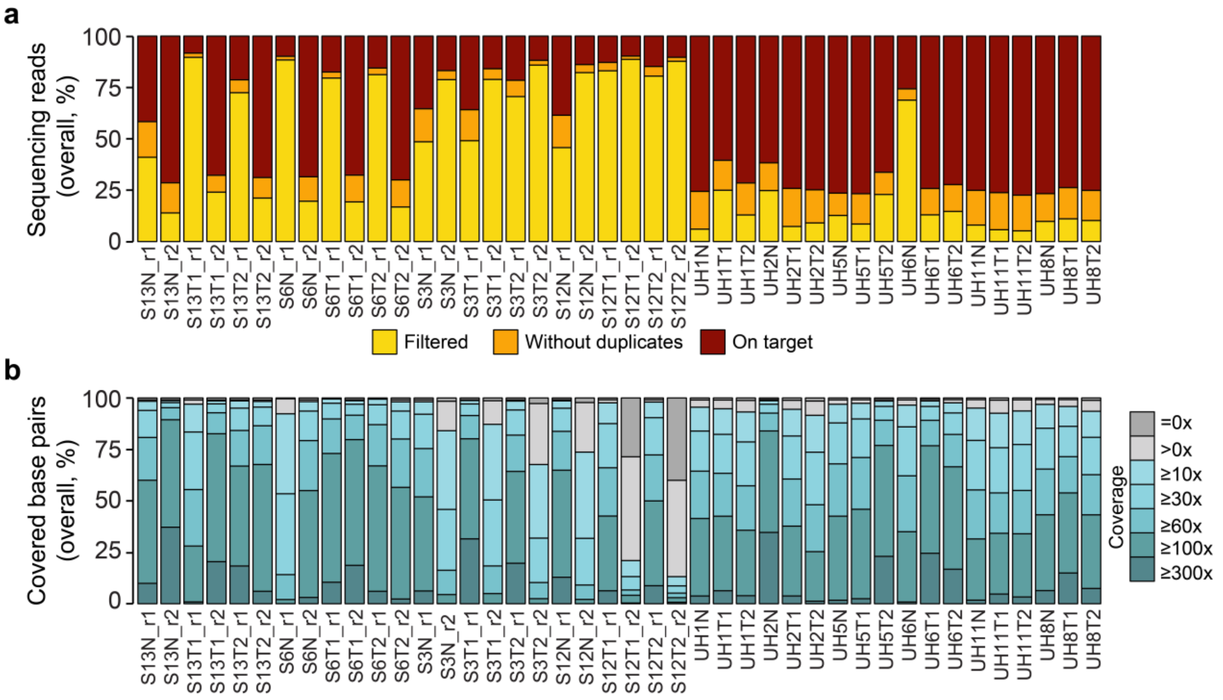

a, Percentage of reads retained at each filtering steps over the total number of aligned sequencing reads. Two distinct exome sequencing rounds (r1 and r2) were performed for both tumours of patients S13, S6, S3 and S12.

b, Percentage of targeted base pairs that were sequenced at different depth of coverage.

Supplementary Figure 3. Variant calling pipelines

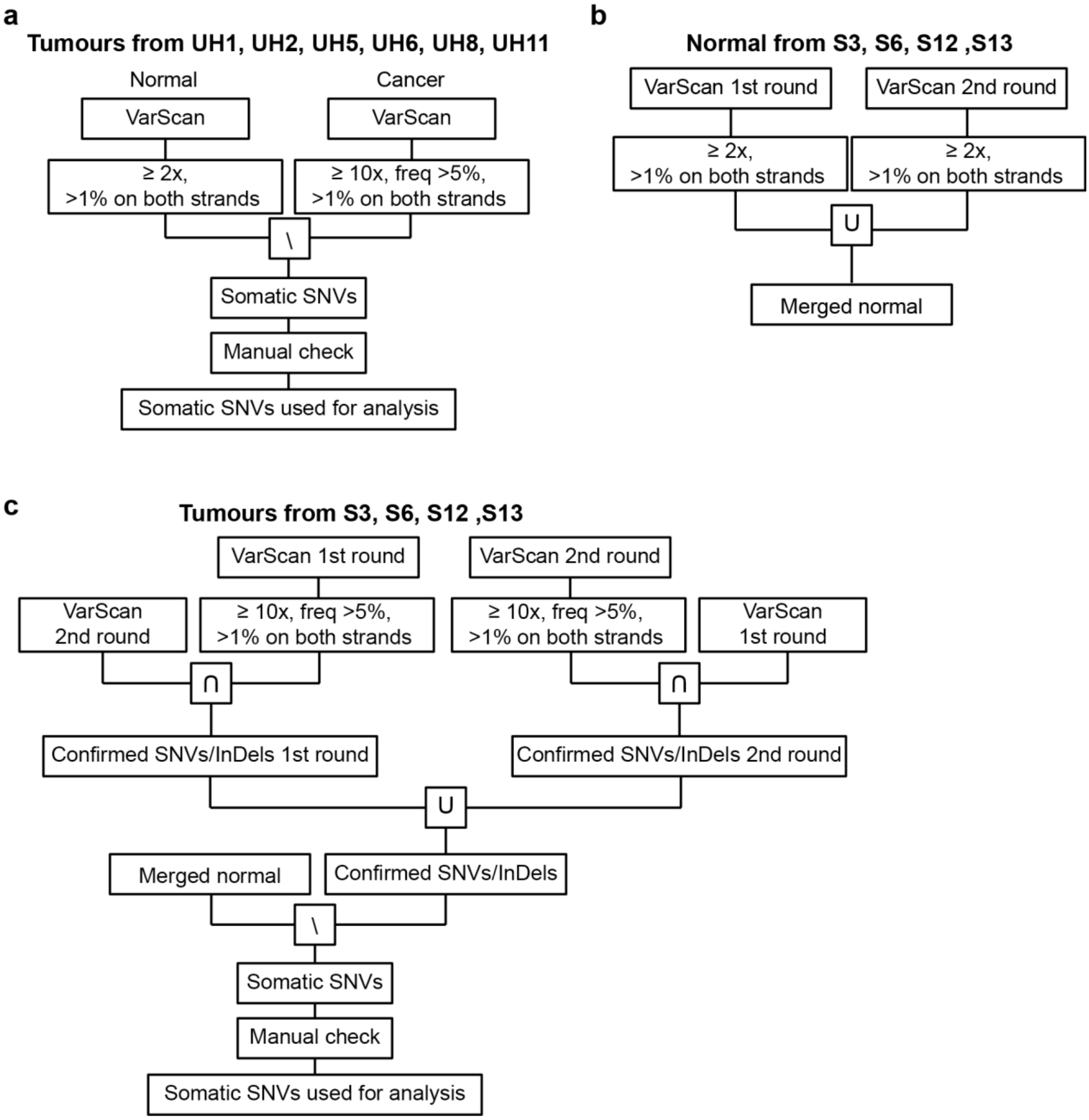

a, Identification of somatic mutations in tumours of patients UH1, UH2, UH5, UH6, UH8 and UH11. Mutations were called and filtered based on quality metrics on tumour and matched normal independently. Somatic mutations were then identified as tumour-specific mutations and all manually inspected.

b, Identification of germline mutations in normal samples of patients S13, S6, S3, and S12 that underwent two rounds of whole exome sequencing. Mutations were first called and filtered for quality in each sequencing round and then merged into a single set.

c, Identification of somatic mutations in tumours of patients S13, S6, S3, and S12 that underwent two rounds of whole exome sequencing. Mutations were called and filtered for quality in each experiment independently and retained only if found in the other sequencing round. The two confirmed sets were merged, somatic mutations were identified as tumour-specific mutations and manually inspected.

Supplementary Figure 4. Signatures of somatic and germline mutations

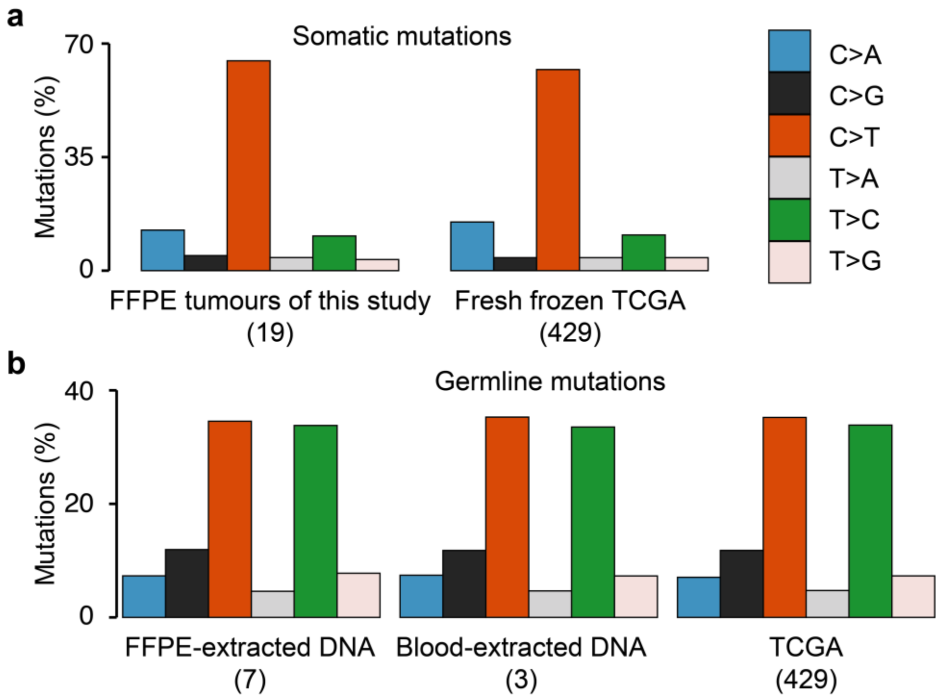

a, Average percentage of base substitutions in FFPE tumours sequenced in this study and fresh frozen colorectal cancers collected from TCGA. The mutational signature is the most prevalent in CRC[1].

b, Average percentage of base substitutions in normal DNA from FFPE blocks, from blood sequenced in this study and from TCGA samples. The mutational signature is in agreement with previous report[2].

Supplementary Figure 5. Coverage of mutated sites in paired lesions

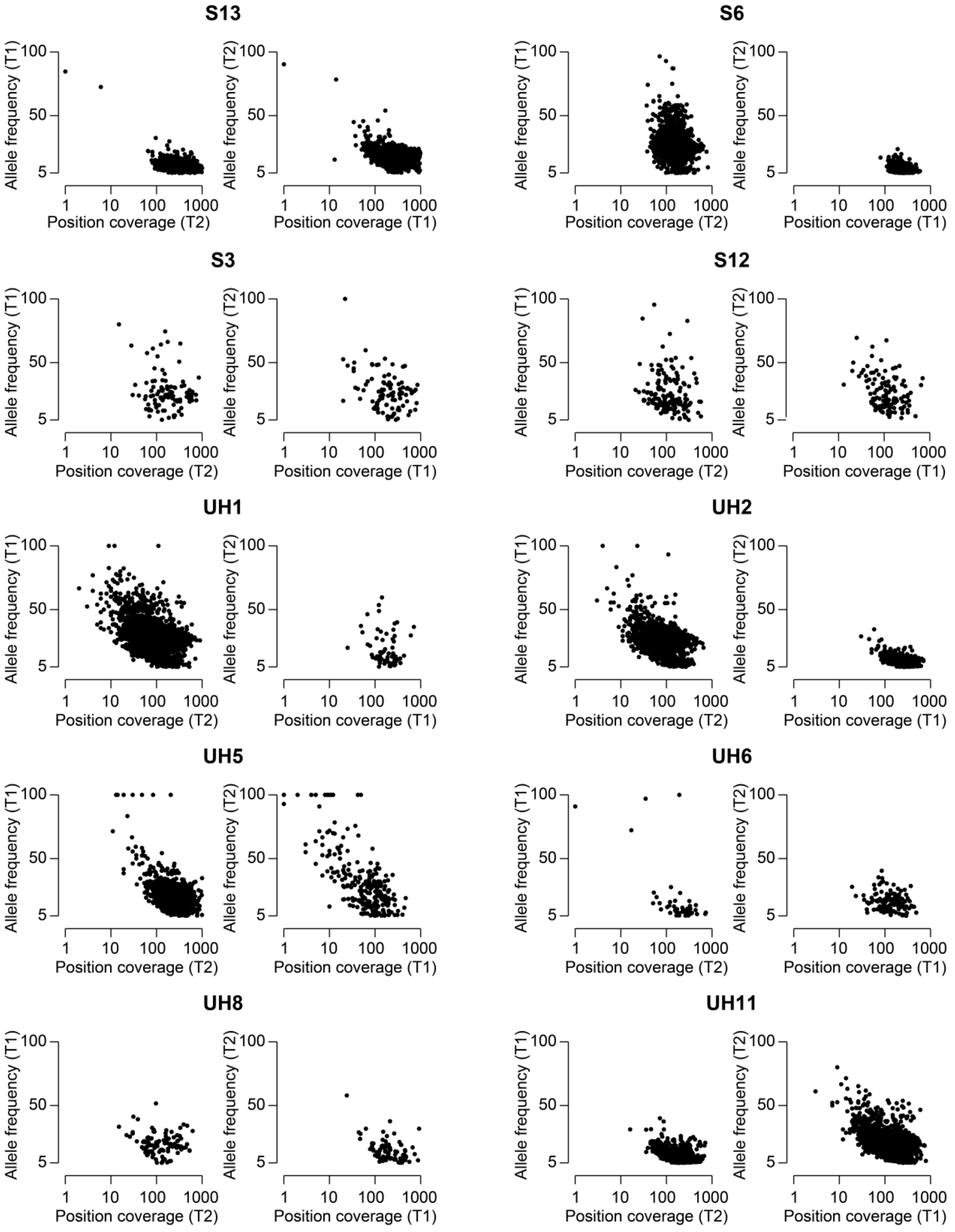

For each patient, reported are the allele frequency of somatic mutations in one tumour and the depth of coverage of the corresponding position in the other tumour.

Supplementary Figure 6. Altered cancer genes in lesions of patients S3, S12, UH5

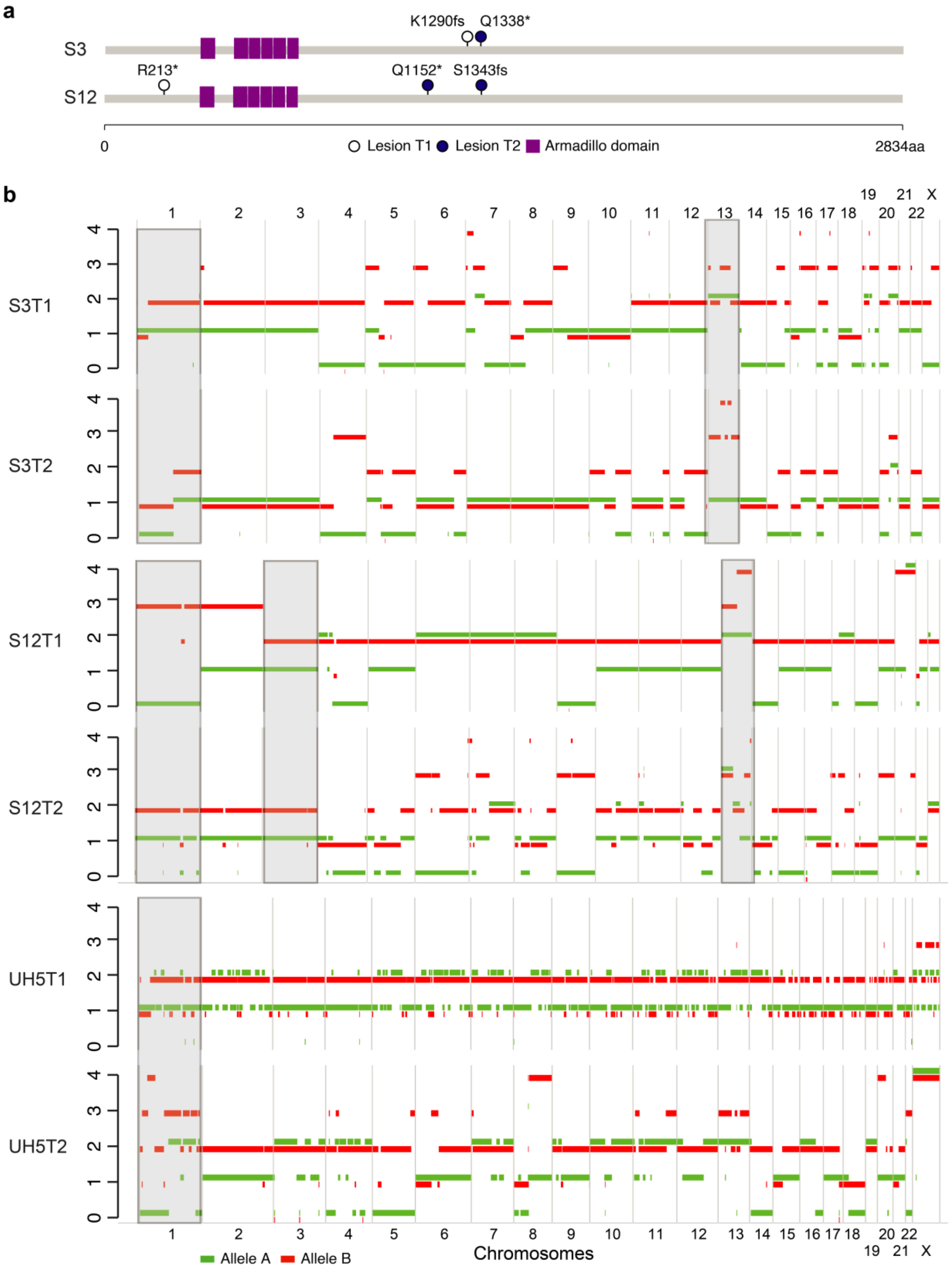

a, Mutations in APC found in tumours of patients S3 and S12. Mutations are indicated according to the Human Genome Variation Society (http://www.hgvs.org/mutnomen).

b, Allele-specific copy number of all assayed loci in tumours of patients S3, S12 and UH5. Alleles with highest copy number (A) and with lowest copy number (B) are shown. Highlighted are chromosomes with shared caner genes between paired lesions of a patient.

Supplementary Figure 7. Allele frequency in whole exome and deep sequencing

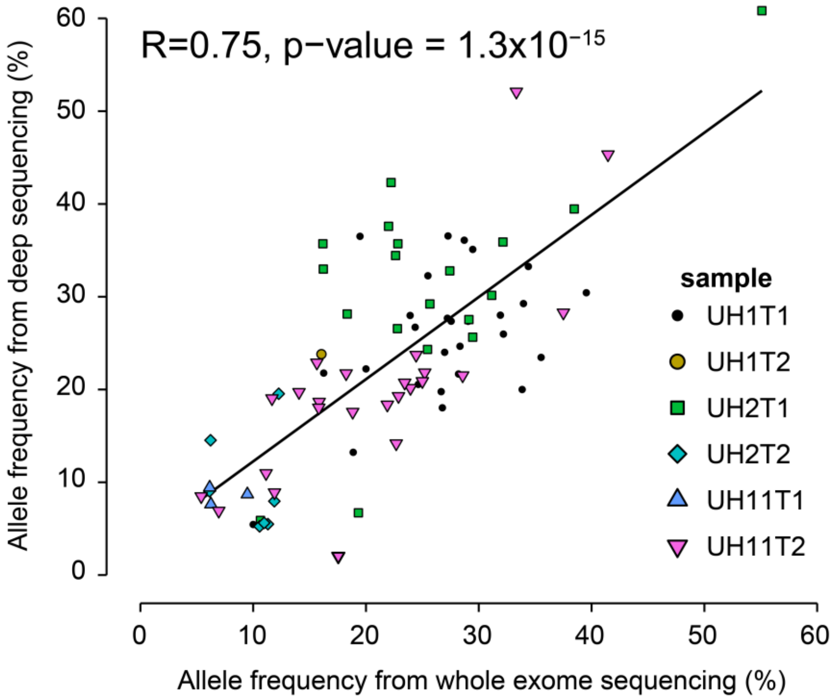

Allele frequencies of somatic mutations in 151 cancer genes in tumours of patients UH1, UH2 and UH11 as detected by whole exome and deep sequencing. Pearson's correlation coefficient (R) and the corresponding p-value are reported.

Supplementary Figure 8. Clone composition of syCRCs

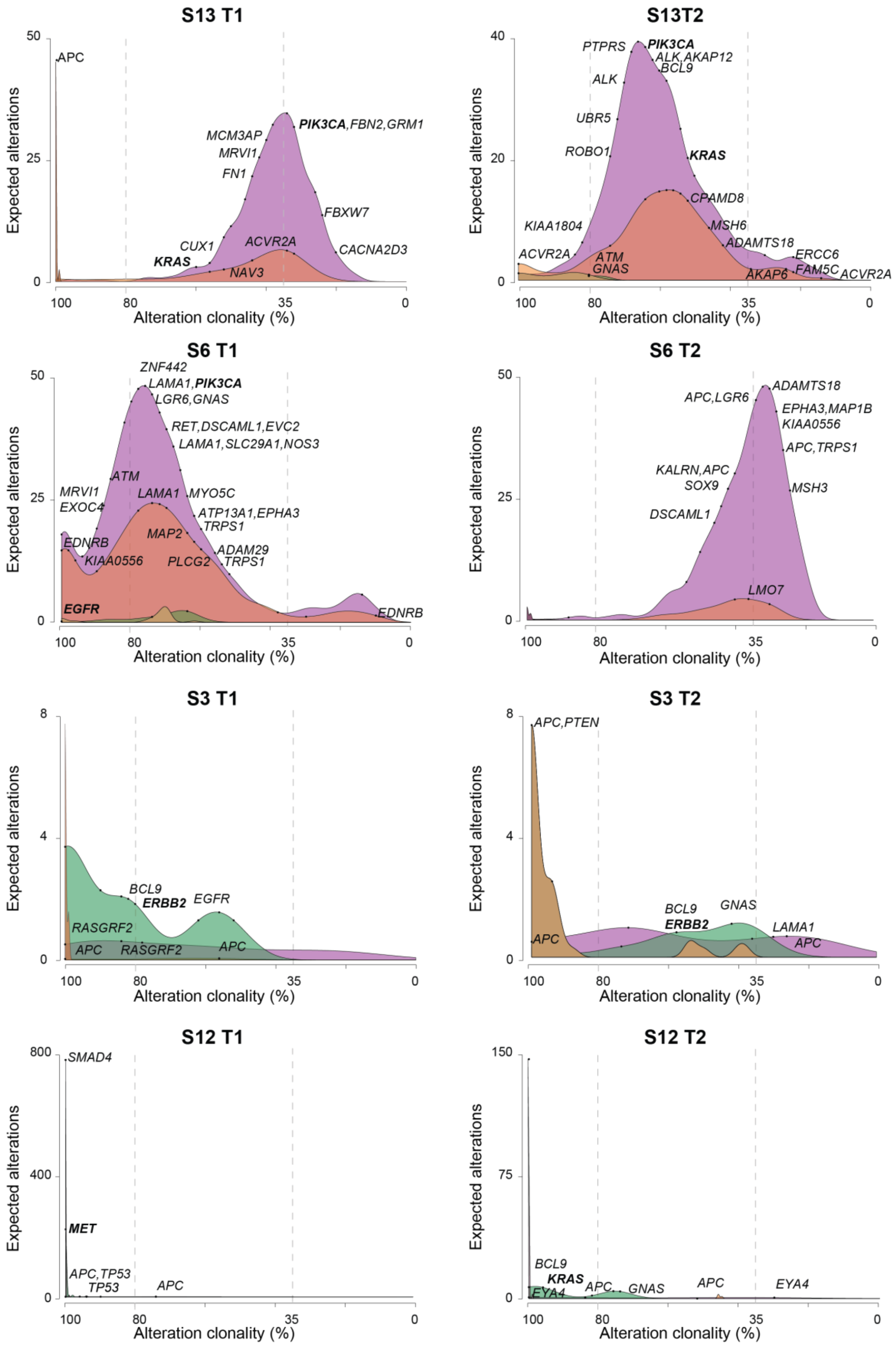

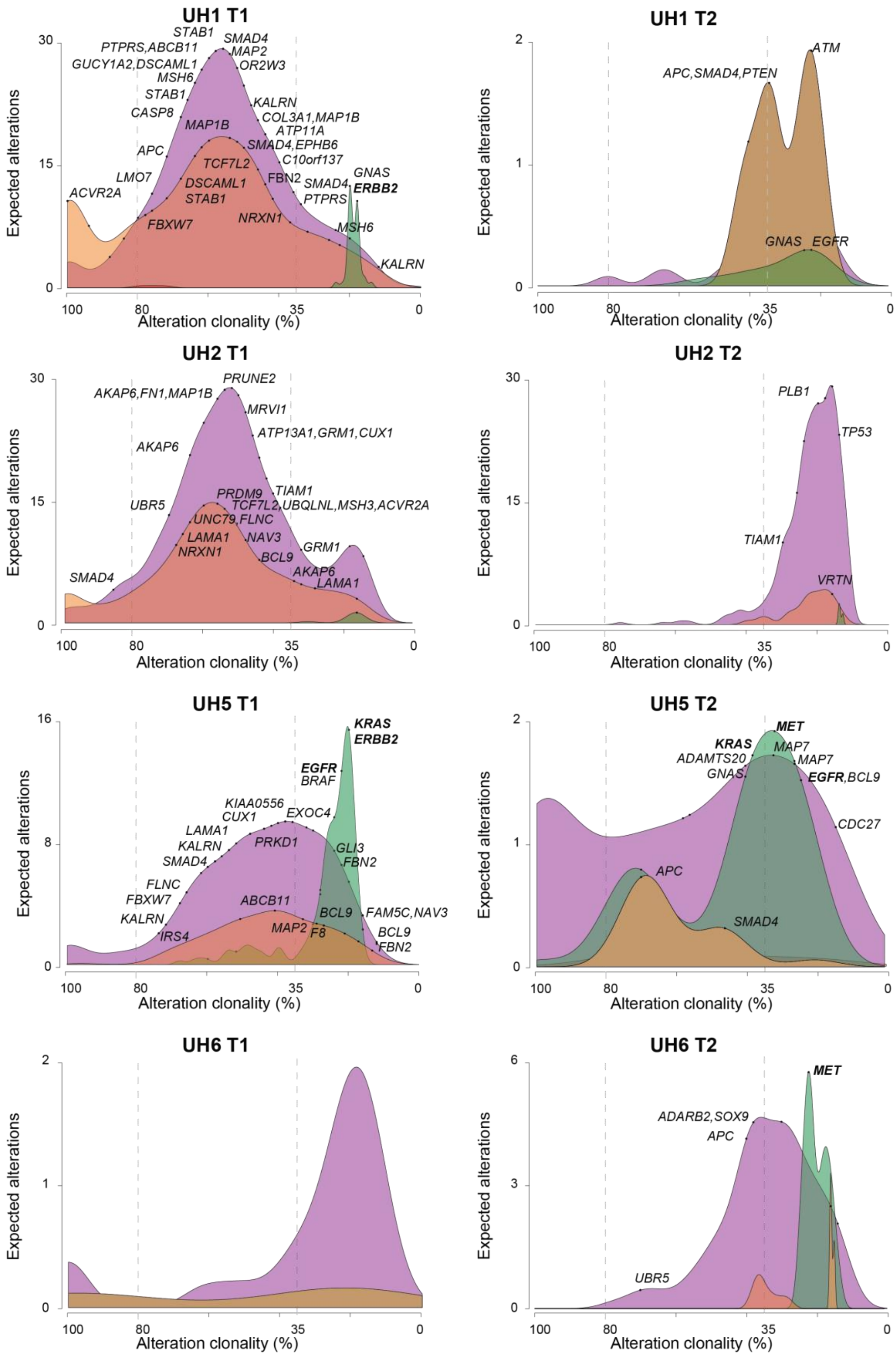
UH1 T1
UH1 T2
UH2 T1
UH2 T2
UH5 T1
UH5 T2
UH6 T1
UH6 T2
Expected alterations
Alteration clonality (%)
100
80
35
0
STAB1
SMAD4
MAP2
OR2W3
PTPRS,ABCB11
GUCY1A2,DSCAML1
MSH6
KALRN
CASP8
COL3A1,MAP1B
MAP1B
ATP11A
APC
SMAD4,EPHB6
TCF7L2
C10orf137
LMO7
FBN2
GNAS
DSCAML1
SMAD4
ERBB2
ACVR2A
PTPRS
NRXN1
FBXW7
MSH6
KALRN
ATM
APC,SMAD4,PTEN
GNAS
EGFR
PRUNE2
AKAP6,FN1,MAP1B
MRVI1
ATP13A1,GRM1,CUX1
AKAP6
TIAM1
PRDM9
UBR5
TCF7L2,UBQLNL,MSH3,ACVR2A
UNC79,FLNC
LAMA1
NAV3
NRXN1
GRM1
BCL9
AKAP6
SMAD4
LAMA1
PLB1
TP53
TIAM1
VRTN
KRAS
ERBB2
EGFR
BRAF
KIAA0556
CUX1
EXOC4
LAMA1
KALRN
PRKD1
GLI3
SMAD4
FBN2
FLNC
FBXW7
ABCB11
BCL9
FAM5C,NAV3
KALRN
IRS4
MAP2
F8
BCL9
FBN2
MET
MAP7
ADAMTS20
MAP7
GNAS
EGFR,BCL9
CDC27
APC
SMAD4
ADARB2,SOX9
APC
UBR5

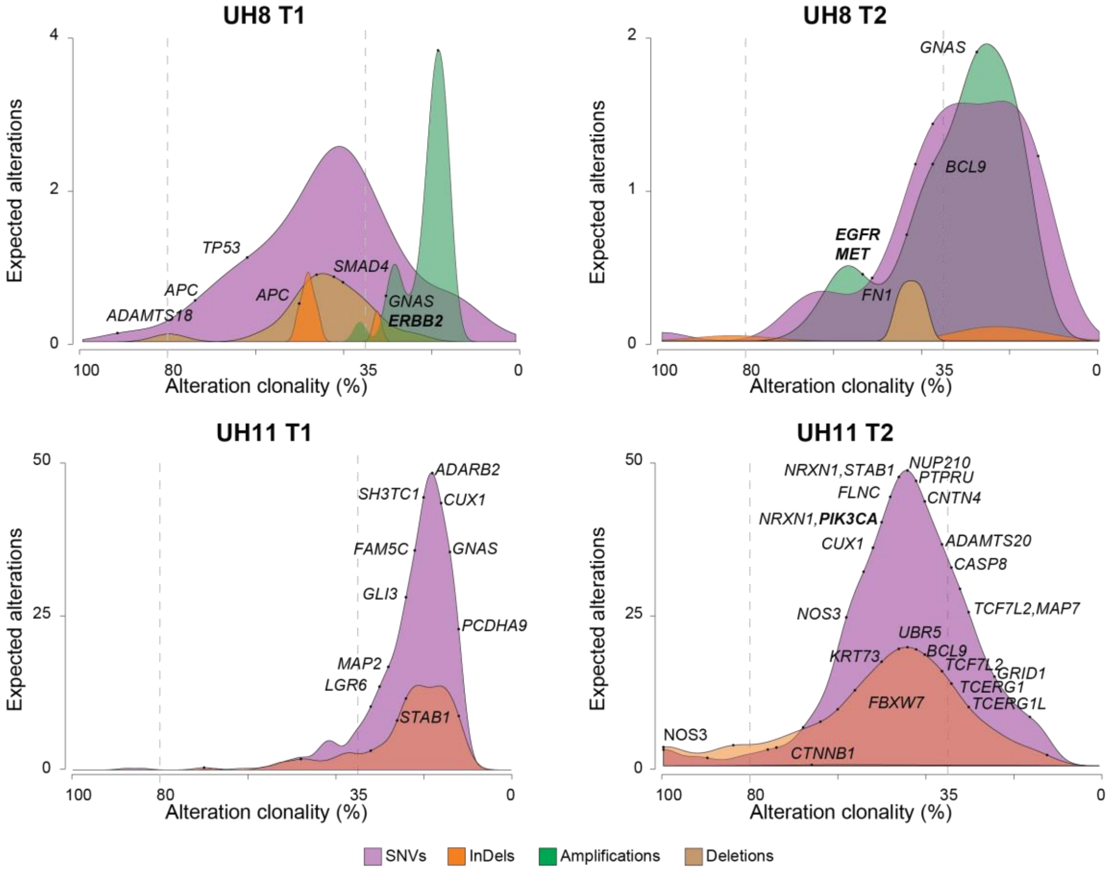

For each lesion of all patients, the density distribution of clonality for each type of alteration (SNVs, InDels, amplifications and deletions) is reported. Dots represent driver alterations in known colorectal cancer genes.

Supplementary Figure 9. Clone composition estimated with our method and with CCF

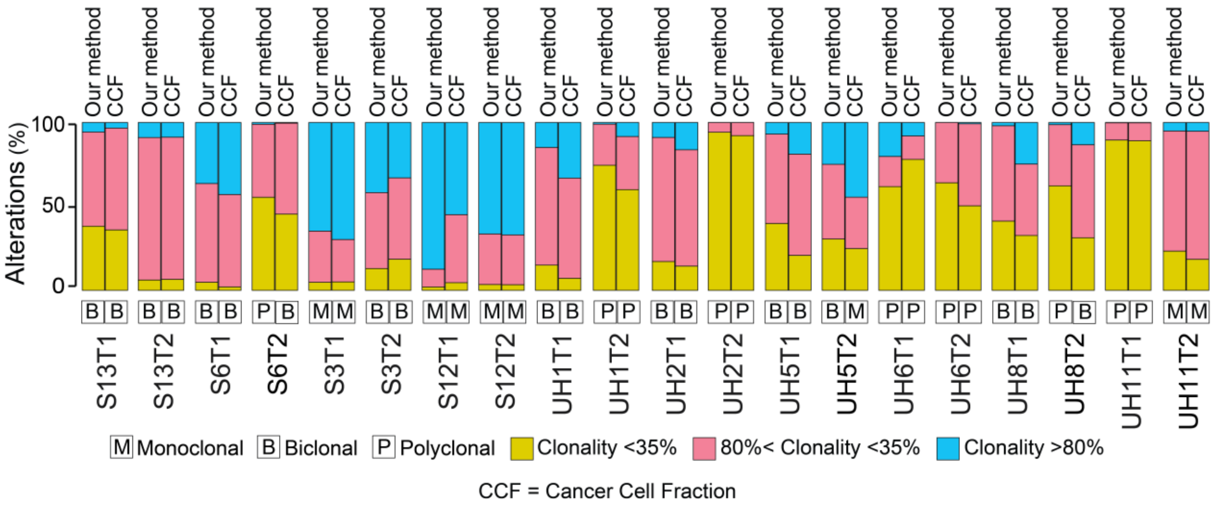

In each tumour, alteration clonality was measured with our method and considering the Cancer Cell Fraction (CCF)[3]. From these measures the corresponding clone composition was derived. To apply CCF, we first estimated the cancer cell fraction of all somatic mutations in each tumour and then used these measures to calculate the clone composition. In 17 out of 20 tumours, we find highly comparable clonality estimations resulting in identical clone composition. In three cases (S6T2, UH5T2 and UH8T2) our method detected slightly higher fractions of subclonal mutations. This is likely due to the fact that we also account for the clonality of copy number variant regions, which are instead excluded in CCF.

Supplementary Figure 10. Alteration clonality of actionable genes in syCRCs

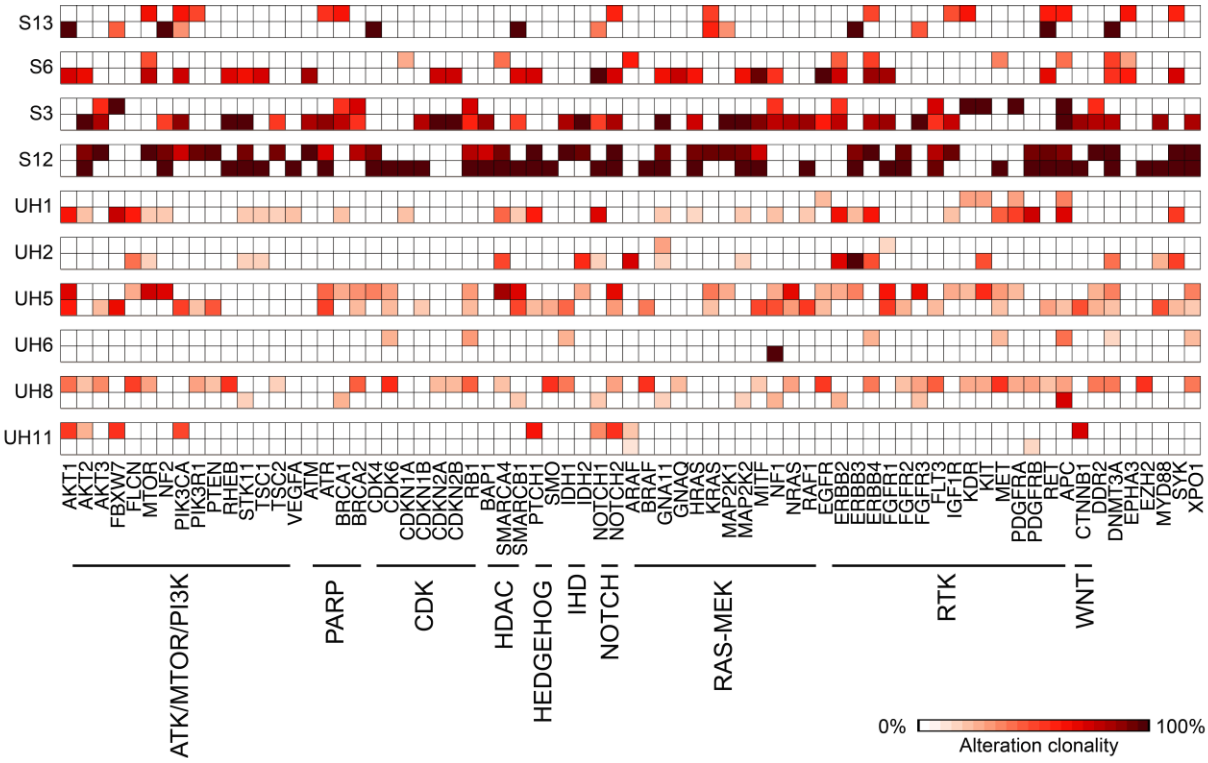

Clonality of alterations in actionable genes relevant in cancer therapy (see main text). Genes are grouped according to pathways. Tumours of patients S12 and S3 showed several clonal CNVs in chromosomes 1 and 3 (Supplementary Fig. 6).

Supplementary Figure 11. Clinical features of syCRC and soCRC patients

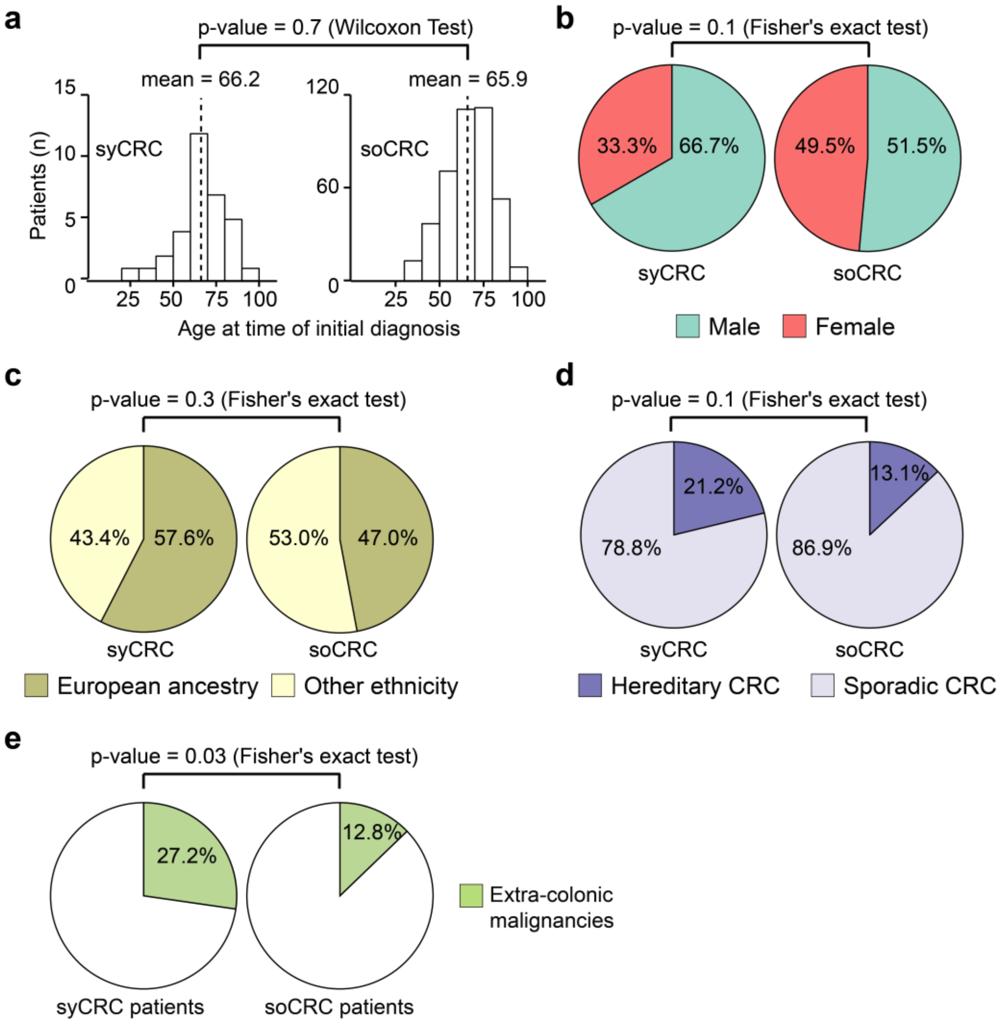

a, Distributions of age at initial diagnosis of syCRC (33) and soCRC (406) patients.

b-e Composition of syCRC (33) and soCRC (406) cohorts in terms of gender, ethnicity, type of colorectal cancer, and presence of extra-colonic malignancies.

Supplementary Figure 12. Neutrophils and lymphocytes in syCRCs and soCRCs

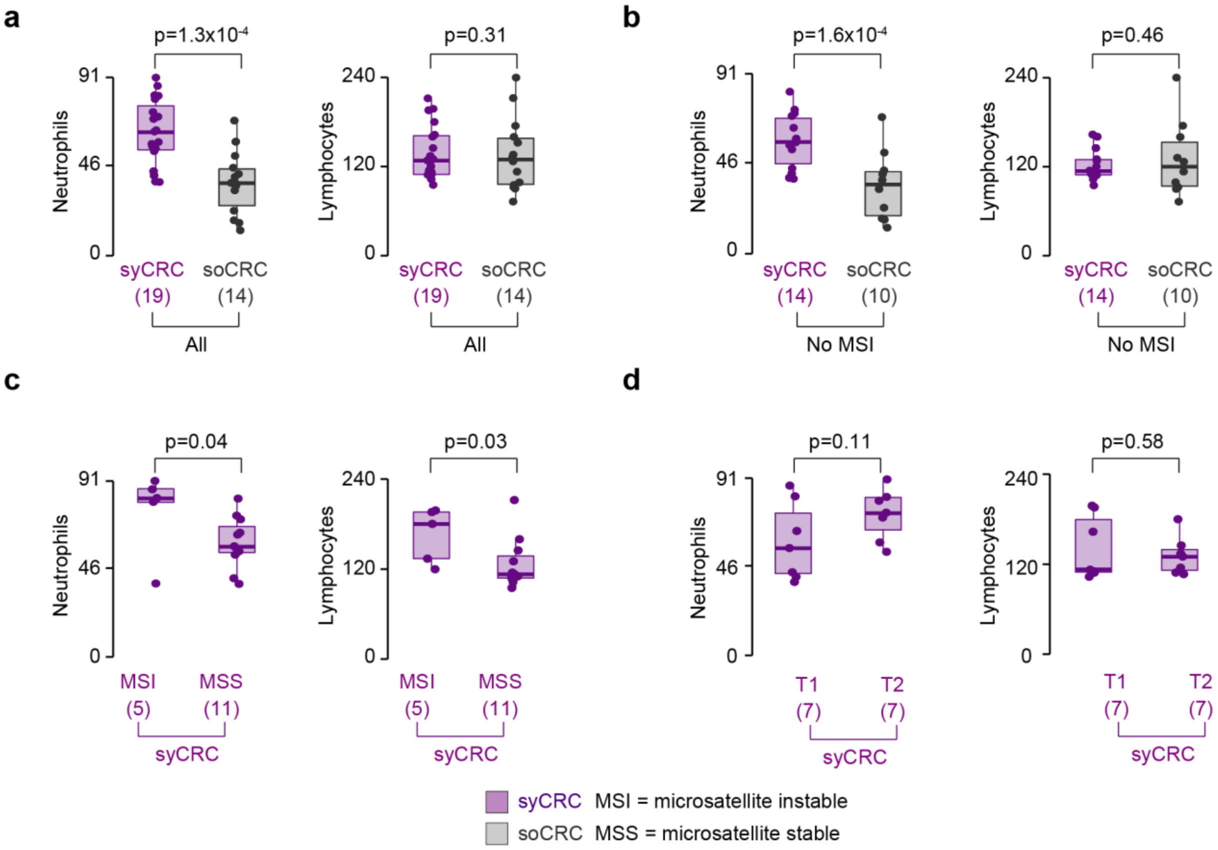

Comparison of neutrophils and lymphocyte between syCRCs and soCRCs overall (a) and considering MSI and MSS samples separately (b). Comparison of neutrophils and lymphocyte between MSI and MSS (c) and T1 and T2 (d) syCRCs. Distributions were compared using one-tailed Wilcoxon rank-sum test. Boxes represent to the 25th and 75th percentiles of each distribution, and the horizontal bars correspond to the median value. Whiskers indicate the values observed within up to 1.5 times the interquartile range above and below each box.

Supplementary Figure 13. Permutation tests on gene expression

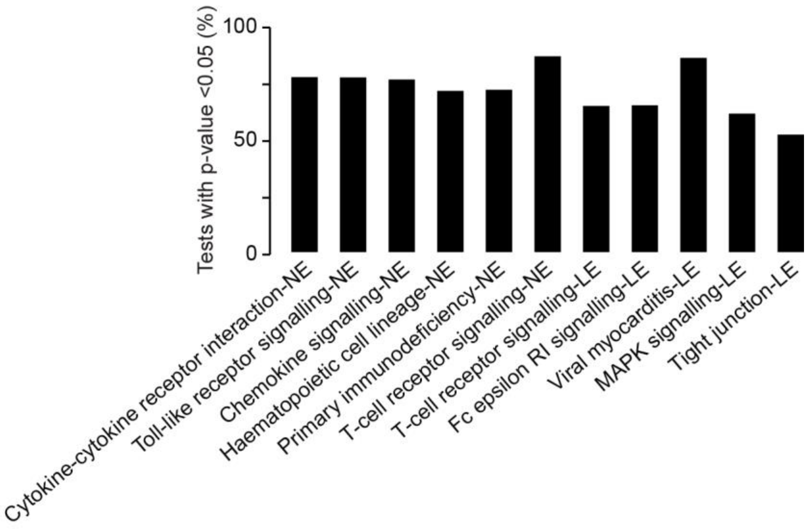

Percentage of statistically significant comparisons between 14 syCRCs and 14 randomly extracted soCRCs (Fisher’s exact text). Only results for the KEGG gene sets that were enriched in not expressed and lowly expressed genes are shown. NE = not expressed; LE = lowly expressed.

Supplementary Figure 14. Expression levels of genes with rare damaging SNPs

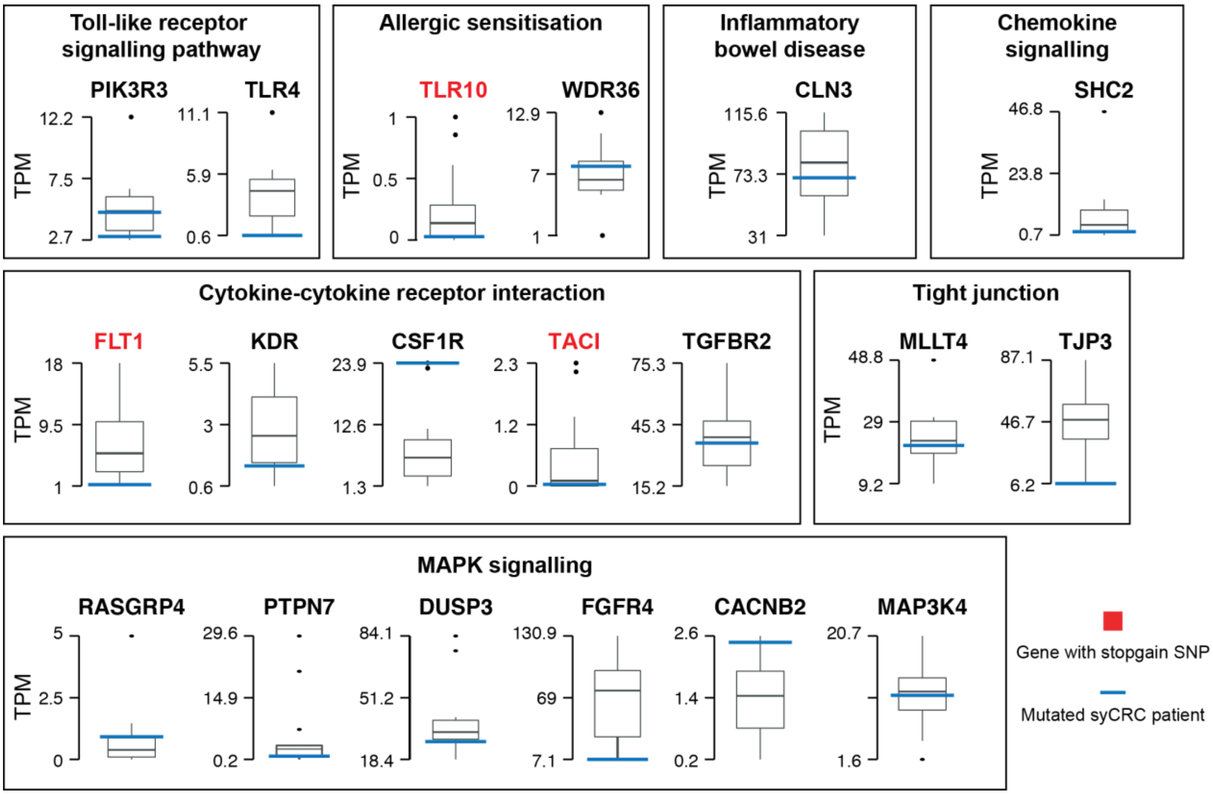

Distribution of expression values (Transcripts per Million, TPM) of mutated and wild-type genes in the pathways enriched in Fig. 3c and Fig. 4h in TCGA syCRCs.

Supplementary Table 1. Clinical information of 20 syCRCs sequenced in the study

Patient ID	Other malignancies	Lesion	Source	Anatomical site	Tumour Stage	Size (cm)	Tumour Content (%)	Microsatellite instability		MMR immune histochemestry
								Markers	Unstable	
S13	Papillar Carcinoma	T1	FFPE	Ascending Colon	T3	4	49	BAT-26, BAT-25, NR-21, NR-24, MONO-27	5	Loss of MLH1
		T2	FFPE	Ascending Colon	T3	2.6	60	BAT-26, BAT-25, NR-21, NR-24, MONO-27	5	Loss of MLH1
S6	-	T1	FFPE	Ascending Colon	T3	4	66	BAT-26, BAT-25, NR-21, NR-24, MONO-27	5	Loss of MSH2
		T2	FFPE	Ascending Colon	T3	3	41	BAT-26, BAT-25, NR-21, NR-24, MONO-27	5	Loss of MSH2
S3	-	T1	Fresh frozen	Rectum	T3	3.4	70	BAT-26, BAT-25, NR-21, NR-24, MONO-27	0	Not investigated
		T2	FFPE	Descending Colon	T1	2.1	77	BAT-26, BAT-25, NR-21, NR-24, MONO-27	0	Not investigated
S12	-	T1	FFPE	Descending Colon	T3	2.5	62	BAT-26, BAT-25, NR-21, NR-24, MONO-27	0	Not investigated
		T2	FFPE	Descending Colon	T2	4	54	BAT-26, BAT-25, NR-21, NR-24, MONO-27	0	Not investigated
UH1	-	T1	FFPE	Caecum	T3	8	≥90	Not investigated		None detected
		T2	FFPE	Ascending Colon	T2	1.3	≥90	Not investigated		None detected
UH2	Breast Cancer	T1	FFPE	Ascending Colon	T2	2.5	≥90	Not investigated		None detected
		T2	FFPE	Transverse Colon	T3	3.2	≥90	Not investigated		None detected
UH5	-	T1	FFPE	Hepatic Flexure	T2	2.2	≥90	Not investigated		Loss of MLH1, PMS2
		T2	FFPE	Rectum	T2	2.3	≥90	Not investigated		Loss of MLH1, PMS2
UH6	-	T1	FFPE	Rectum	T3	5	≥90	Not investigated		None detected
		T2	FFPE	Rectum	T3	4.5	≥90	Not investigated		None detected
UH8	-	T1	FFPE	Descending Colon	T1	1.5	≥90	Not investigated		None detected
		T2	FFPE	Descending Colon	T1	2	≥90	Not investigated		None detected
UH11	-	T1	FFPE	Caecum	T2	3	≥90	Not investigated		Loss of MLH1, PMS2
		T2	FFPE	Transverse Colon	T3	2.5	≥90	Not investigated		Loss of MLH1, PMS2

For each patient, reported are other malignancies (if any), the preservation method of the tissue and pathological information on both lesions. MMR: mismatch repair proteins.

Supplementary Table 2. Sequencing statistics for whole exome sequencing in 20 tumour and 10 normal samples

Patient ID	Sample Type	Sequencing round	Sequenced Gbp	Aligned Gbps	Gbps after removal of duplicates	On Target Gbps	Mean coverage overall (reads)	Mean coverage on cancer genes (reads)
S13	N	1	35.31	22.09	13.03	7.78	152	137
	T1		57.23	55.62	5.76	4.12	80	243
	T2		54.00	52.32	14.42	9.95	194	76
	N	2	23.47	22.67	19.52	13.80	270	221
	T1		18.82	17.97	13.65	10.77	210	182
	T2		13.25	12.62	9.95	7.60	148	153
S6	N	1	32.09	27.08	3.15	1.84	36	38
	T1		63.73	60.69	12.41	8.65	170	129
	T2		68.97	64.06	12.02	7.57	148	134
	N	2	11.74	10.58	8.50	6.27	123	210
	T1		20.32	17.38	14.03	10.31	201	151
	T2		12.09	10.20	8.49	6.27	122	128
S3	N	1	37.88	22.14	11.39	6.59	129	117
	T1		46.16	44.54	22.69	13.80	271	37
	T2		58.77	56.73	16.71	10.30	202	270
	N	2	14.65	12.50	2.65	1.82	36	24
	T1		17.02	14.20	3.00	1.95	38	195
	T2		14.61	12.98	1.83	1.33	26	26
S12	N	1	26.48	25.70	13.95	8.62	168	165
	T1		54.79	52.74	8.91	6.03	118	28
	T2		54.64	52.58	10.25	6.96	136	103
	N	2	13.16	11.30	2.01	1.35	26	22
	T1		13.46	9.35	1.06	0.79	15	119
	T2		9.83	6.90	0.85	0.62	12	20
UH1	N	1	8.78	8.28	7.78	5.28	104	106
	T1		22.44	11.16	8.37	5.75	113	115
	T2		8.53	8.00	6.97	4.88	96	98

UH2	N	1	27.45	26.00	19.55	13.34	261	277
	T1		22.97	8.04	7.46	5.03	99	100
	T2		6.17	5.83	5.30	3.75	74	75
UH5	N	1	8.28	7.89	6.89	5.11	100	109
	T1		8.89	8.55	7.82	5.48	107	117
	T2		23.09	19.22	14.82	10.78	211	226
UH6	N	1	24.84	21.84	6.81	4.48	88	96
	T1		13.06	9.98	9.00	6.09	119	125
	T2		18.04	14.74	12.59	9.08	178	194
UH8	N	1	9.16	8.87	8.00	5.83	114	114
	T1		13.80	13.13	11.68	7.97	156	156
	T2		10.07	9.59	8.61	5.92	116	115
UH11	N	1	7.32	6.92	6.37	4.33	85	86
	T1		8.09	7.60	7.17	4.87	96	94
	T2		7.51	7.15	6.78	4.68	92	90

For each sample, sequenced, aligned, aligned after removal of PCR duplicates, and on target Giga base pairs (Gbps) are provided. The mean depth of coverage considering on target reads across the all exome and on cancer genes is also provided.

Supplementary Table 3. Quality controls on variant calling

Patient ID	Lesion	Sequencing round	VarScan	≥10x, freq>5%, >1% on both strands	Confirmed in the other	Round overlap (%)	Union	Somatic SNVs/InDels	Somatic SNVs	SNVs confirmed with MuTect (%)	Somatic InDels	Confirmed with Strelka (%)
S13	T1	1	785,500	42,657	38,268	90%	43,466	1,143	928	93%	215	74%
	T1	2	438,492	50,055	42,836	86%						
	T2	1	409,217	48,714	42,245	87%	43,861	1,659	1,153	95%	506	69%
	T2	2	447,745	49,338	42,510	86%						
S6	T1	1	615,615	45,314	40,117	89%	55,495	2,891	1,842	99%	1,049	100%
	T1	2	585,487	29,800	27,028	91%						
	T2	1	718,121	44,431	38,489	87%	49,606	1,056	949	98%	107	100%
	T2	2	820,005	22,345	19,997	89%						
S3	T1	1	211,346	45,314	40,117	89%	40,439	101	99	91%	2	74%
	T1	2	396,122	29,800	27,028	91%						
	T2	1	352,704	44,431	38,489	87%	38,711	102	101	89%	1	67%
	T2	2	315,877	22,345	19,997	89%						
S12	T1	1	601,073	41,003	21,682	53%	21,840	158	158	89%	0	100%
	T1	2	114,876	5,754	4,287	75%						
	T2	1	620,773	42,268	17,334	41%	17,427	121	116	88%	5	80%
	T2	2	82,795	3,565	2,554	72%						
UH1	T1	1	886,994	42,996	-	-	-	2,740	1,654	94%	1,086	61%
	T2	1	818,666	37,652	-	-	-	63	60	90%	3	33%
UH2	T1	1	783,515	41,885	-	-	-	1,739	1,174	94%	565	78%
	T2	1	817,796	37,146	-	-	-	531	469	76%	62	89%
UH5	T1	1	755,797	43,040	-	-	-	1,154	840	89%	314	90%
	T2	1	463,864	44,133	-	-	-	251	242	55%	9	56%
UH6	T1	1	542,675	41,813	-	-	-	51	49	73%	2	100%
	T2	1	690,682	42,479	-	-	-	136	130	90%	6	100%
UH8	T1	1	566,248	40,930	-	-	-	103	98	85%	5	80%
	T2	1	605,477	38,751	-	-	-	84	80	83%	4	50%
UH11	T1	1	890,739	46,823	-	-	-	985	720	87%	265	80%

	T2	1	795,024	49,214	-	-	-	2,164	1,468	93%	696	73%

For each tumour, reported are the number of mutations at each step of the variant calling pipeline, as described in Supplementary Fig. 3. Tumors of patients S13, S6, S3 and S12 underwent two rounds of sequencing and the overlap between the two rounds is shown. The second round of S12T1 and S12T2 had lower coverage (Supplementary Fig. 2) and therefore the overlap d is lower. Finally, the percentage of somatic SNVs and InDels that were confirmed with MuTect and Strelka are also reported.

Supplementary Table 4. Sequencing statistics for deep sequencing of a cancer gene panel in 6 tumour samples

Patient ID	Sample Type	Sequenced Gbps	Aligned Gbps	Gbps after removal of duplicates	On Target Gbps	Mean coverage (reads)
UH1	T1	1.95	1.03	0.58	0.21	265
	T2	1.94	0.54	0.27	0.09	115
UH2	T1	1.92	0.72	0.37	0.12	157
	T2	1.95	1.73	0.73	0.24	307
UH11	T1	1.27	1.18	0.73	0.26	335
	T2	1.99	1.86	1.06	0.40	505

For each sample, sequenced, aligned, aligned after removal of PCR duplicates, and on target Giga base pairs (Gbps) are provided. The mean depth of coverage considering on target reads on 151 cancer genes is provided.

Supplementary Table 5. Results of the Sanger sequencing validation

Coordinate (Hg19)	Ref	Var	Gene	Mutation type	SNP ID (dbSNP138)	Mutated sample (ID)	Forward primer sequence	Reverse primer sequence	Amplicon size (bp)	Confirmed
2:148683685	T	-A	ACVR2A	frameshift deletion	-	UH1T1	GAGGAAATTGGCCAGCATCC	TGCAGAAGAAAGAGAAATGTGC	190	TRUE
5:112128191	C	T	APC	Stopgain	-	UH8T1	CCTGAGCTTTTAAGTGGTAGCC	GCTTCTGTTGCTTGGGACTG	171	TRUE
5:112175754	T	-A	APC	frameshift deletion	-	UH8T1	CATGCCACCAAGCAGAAGTA	CACTCAGGCTGGATGAACAA	233	TRUE
5:112175303	C	T	APC	Stopgain	-	S3T2	CTGCCACTTGCAAAGTTTCT	AAACATGAGTGGGGTCTCCT	416	TRUE
5:112175215	A	+A	APC	frameshift insertion	-	S3T1	CCATTCCTACAGAAGGCAGA	TGAGGTGAATCAAAAGCAAA	466	TRUE
5:112154724	G	A	APC1	nonsynonymous	rs377665107	UH1T1	AAACTCATTTGGCCCACAGG	GCTGGATGAGGAGAGGAAGA	161	TRUE
5:112175211	T	-AAAAG	APC2	frameshift deletion	-	UH1T2	ATCAGACGACACAGGAAGCA	TGCCTGGCTGATTCTGAAGA	178	TRUE
5:112176026	A	-T	APC3	frameshift deletion	-	UH5T2	ACCAAGAGAAAGAGGCAGAAA	GTACACAGGCAGCTGACTTG	203	FALSE
1:27105930	T	+G	ARID1A	frameshift insertion	rs1057192	UH6T1	GTGCAGGAGTTTGACAGTGG	TCTGTTGTCCCTGGTGTACC	179	FALSE
20:31657760	C	T	BPIFB3	nonsynonymous	-	UH6T2	ACAGATGGATGGAGGCAGAC	GATGCTCAAGAAACGGGAGG	238	TRUE
11:119170274	G	A	CBL	nonsynonymous	-	UH8T2	AGAGGCCTCCAAAACCATTC	GGCCATCTCGATGTTGTTCT	203	TRUE
16:15131330	G	A	CCDC105	nonsynonymous	rs35352238	UH6T1	ACTCTGTCTTCTCCTCCCCT	GTCTGGAGGAGGCGTTTTCT	207	FALSE
3:41266124	A	G	CTNNB1	nonsynonymous	rs121913412	UH11T2	ATGGAGTTGGACATGGCCAT	TCCACATCCTCTTCCTCAGG	152	TRUE
21:38858865	C	T	DYRK1A	Stopgain	-	UH2T1	GGCATATGATCGTGTGGAGC	AAGTTTTGGTGTGGGTTGGG	190	TRUE
6:116783330	G	A	FAM26F	nonsynonymous	-	UH6T1	GCCTGGTCTTCTTGCTGGT	CACTCGTAAAAGGCGCCC	223	TRUE
4:153244092	G	A	FBXW7	nonsynonymous	-	UH5T1	GGGCAGGGAGTATATCGTCT	AGTCACATTGGAGAGTGGGG	197	TRUE
2:216274342	G	A	FN1	nonsynonymous	-	UH8T2	CATCTCCCTCCTCACTCAGC	TAAAATGATGTTGGCGACGA	240	TRUE
22:36889782	C	A	FOXRED2	stopgain	-	UH5T1	TGGGAGCTTAAGTTACCTGCA,	CTCTCCTCCCCACAGAACAG	202	TRUE
7:151845523	T	-A	MLL3	frameshift deletion	-	UH11T1	TAGCAATCTGTCGCACCTCA	TGCCACAGATTTCGATGCAC	190	TRUE
2:48028135	C	T	MSH6	stopgain	rs63750563	UH1T1	GTAGGAACCGTTACCAGC	TCCCTCCGTTCTTCAGCATT	160	TRUE
2:211179765	A	-T	MYL1	frameshift	-	UH5T2	CTTCTTTGGGTTTGGCTGGG	ACCACCACTCCTCTTCCAAG	192	TRUE
12:57114417	T	+G	NACA	frameshift insertion	-	UH1T1	GCAAGATGAGAGCCCAGAGA	ATCGCTTCCCCTCAAGTCAA	209	TRUE
7:150693568	G	-C	NOS3	frameshift deletion	-	UH11T2	CTGTCCTGACCTTTGCACTC	CGGGAAGCTGTCACCTCTTA	194	TRUE
5:102260714	C	T	PAM	nonsynonymous	-	UH5T2	CCCATCACCTCCCACCATG	AGCTGAACATAAGACCACCCA	204	TRUE
12:133233939	T	C	POLE	nonsynonymous	-	UH11T2	ACCCATCAGAGAGAGACCCT	CTGTTCTCTGTGCCCGTTTC	188	TRUE
9:33798075	T	C	PRSS3;PRSS3	nonsynonymous	-	S13T2	CTCACCTGCCGTCATCAATG	GGCATGCTTCGTTCTGGAAA	177	TRUE
18:48573591	A	G	SMAD4	nonsynonymous	-	UH1T1	ACCCAAGACAGAGCATCAAAG	TAAGGTTAAGGGCCCCAACG	152	TRUE

18:48604835	T	C	SMAD4	stoploss	-	UH2T1	AGACAAGGTGGAGAGAGTGA	TGAAGCCTCCCATCCAATGT	170	TRUE
18:48591919	G	A	SMAD4	nonsynonymous	rs377767347	UH5T1	GCTCCTGAGTATTGGTGTTCC	ACCTTGCTCTCTCAATGGCT	187	TRUE
17:7578457	C	T	TP53	nonsynonymous	-	UH8T1	CTCCGTCATGTGCTGTGACT	GTTTCTTTGCTGCCGTCTTC	221	TRUE
17:7578433	G	C	TP53	stopgain	-	S12T1	CCCAGTTGCAAACCAGAC	CACTTGTGCCCTGACTTTC	442	TRUE
6:139581469	G	A	TXLNB	stopgain	-	UH2T2	ACAACTTGCTCAGAGTGTCTC	CAGAAGCTGGTGGATGCAAA	153	TRUE
8:103283450	G	A	UBR5	stopgain	-	UH6T2	CTGCTCGCAAACCACTACTC	ACACAGATGCTAGGGAAGGA	227	TRUE
17:5036224	C	G	USP6	nonsynonymous	-	S13T1	AGAGCCCAAGACTCAGCATC	GCCTCTCCCTCCACACATTA	181	TRUE
4:1980558	G	-C	WHSC1	frameshift deletion	-	UH1T1	TTCATTTTGCCACCTCTGCC	TTTGCCCTCTGTGACTCTCC	217	TRUE

For each of the 35 mutations, genomic coordinates, reference allele (Ref), variant allele (Var), gene symbol, type of mutation, SNP identifier, mutated sample identifier, sequence of PCR primers, amplicon size and validation result are reported.

Supplementary Note 1

To compare the clone composition of paired tumours, we classified them as monoclonal, biclonal and polyclonal based on the clonality of their somatic alterations, measured as described in Fig. 2a and Methods. In each tumour, we counted how many alterations (SNVs, InDels, amplifications and deletions) had clonality >80%; 35%-80%; and <35% and identified the largest group of the three. On average the largest group accounted for 64% of all somatic alterations of a tumour (Fig. 2b).

The two thresholds of clonality (80% and 35%) that were used to define the three groups allowed the identification of three well distinct scenarios:

- If alterations with clonality >80% were the largest group, the majority of cells shared the same (almost clonal) alterations and the tumour could be considered as mostly composed of a dominant clone (*i.e.* monoclonal);
- If alterations with <35% clonality were the largest group, the tumour could be considered as polyclonal because multiple clones co-existed and contributed with a similar share to the tumour mass (for example there were at least three clones each accounting for ~33% of the mass);
- In the intermediate situation where alterations with 35%< clonality <80% were the largest group, the tumour could be considered as biclonal because this range of frequency is compatible with two main clones forming the tumour mass.

It should be noted that the density distributions that are used to infer the peaks of clonality provide only an estimation of the expected number of mutations at each clonality.

Supplementary References

1. Alexandrov LB, *et al.* Signatures of mutational processes in human cancer. *Nature* **500**, 415-421 (2013).

2. Rahbari R, *et al.* Timing, rates and spectra of human germline mutation. *Nat Genet*, (2015).

3. McGranahan N, Favero F, de Bruin EC, Birkbak NJ, Szallasi Z, Swanton C. Clonal status of actionable driver events and the timing of mutational processes in cancer evolution. *Sci Transl Med* **7**, 283ra254 (2015).
